# Supplementary material for: Are carbon emissions trading and green financial instruments synergistic? -Comprehensive quantitative research based on content analysis
Source: PLoS One. 2024 Mar 7;19(3):e0298601. doi: 10.1371/journal.pone.0298601 (PMC10919601; doi:10.1371/journal.pone.0298601)
Supplement: S1 Data — (ZIP) [file pone.0298601.s001.zip › Data/地级市Z_F工作报告.zip!/╦─┤¿/░═╓╨╩╨/└┤╫╘╣1⁄2╓┌║┼í╛┬φ┐╦╩2╛▌═°í┐_2016.docx]

各位代表：

　　现在，我代表市人民政府，向大会报告工作，请予审议，并请市政协委员和其他列席人员提出意见。

　　过去五年工作回顾

　　市三届人大一次会议以来，在市委坚强领导下，在市人大、市政协监督支持下，我们认真贯彻党的十八大和习近平总书记系列重要讲话精神，深入实施省委“三大发展战略”，全面落实市委各项决策部署，团结带领全市人民，主动适应经济新常态，促进发展新跨越，完成了“十二五”主要目标任务，实现了“十三五”良好开局，开创了经济社会发展新局面。

　　经济发展稳中向好。五年里，我们有效应对经济下行压力，陆续出台稳增长政策措施，全市经济逆势攀升，实现了量的增长和质的提升，多项主要指标增速位居全省前列。预计全市地区生产总值539亿元，较2011年增长64.8%;全社会固定资产投资1200亿元，增长2.7倍;社会消费品零售总额287.5亿元，增长93.1%;地方一般公共预算收入44.29亿元，增长2.5倍;城镇居民人均可支配收入26039元，增长70.4%;农村居民人均可支配收入10029元，增长80.4%。一二三产业增加值年均分别增长3.5%、13%、10.1%，三次产业结构由2011年的21.4:42.5:36.1调整为2016年的16.0:47.1:36.9，呈现出一产趋优、二产领跑、三产提升的良好态势。

　　城乡建设加快推进。五年里，我们坚持不懈推进新型城镇化，加快建设四级城镇体系，城镇化率达到39%，城乡面貌发生深刻变化，居民生活舒适度和幸福感显著提升。完成城市总规修编和市县全域规划，建成市规划馆、体育馆、游泳馆和巴城环线西段北段、秦巴大道、巴恩快速通道，城市空间得到拓展。巴城建设提档升级，经开区产业新区、人居新城初具规模，恩阳设立县级行政区、文旅新城已具雏形，中心城区建成区面积53平方公里、人口61万，巴城正式步入中等城市的行列，其中心地位、吸附能力初步彰显。县城建设加快推进，南江县列入全国第三批新型城镇化综合试点区，通江高明、南江红塔、平昌金宝等新区展现新貌，区域承载能力不断增强。启动实施9个全国重点镇、12个省级试点镇和24个市级重点镇建设，建成一批中心村和巴山新居聚居点，487个村达到幸福美丽新村标准，城乡一体化步伐坚实有力。

　　基础设施明显改善。五年里，我们坚持后发也要高点起步，新上了一大批基础设施项目，瓶颈制约有效破除，发展条件得到改善。全市完成交通、水务、电力投资680.63亿元，是上个五年的4倍。巴中恩阳机场开工;巴达铁路和成(都)巴(中)、巴(中)达(州)、巴(中)陕(西)南江段、巴(中)南(充)广(安)高速公路建成投运;新增普通国省干线1147公里;新(改)建农村公路8103公里，初步形成干支配套、互联互通的区域路网。开工红鱼洞和黄石盘水库，结束了我市没有大型水库的历史;建成二郎庙等3座中型水库，15座水库纳入国省规划，在建大中型水库数量全省第一。四川第二座智能化500千伏变电站建成投运，新(扩)建220千伏变电站3座、110千伏变电站9座。“智慧巴中”建设全面推进，通讯移动网络实现全覆盖，城乡4G覆盖面达60%，有线电视数字化率达到99%，可持续发展基础进一步坚实。

　　脱贫攻坚成效初显。五年里，我们扎实推进扶贫攻坚“六大工程”，落实“六个精准”，推进“五个一批”，全面实施17个扶贫开发专项方案，累计减少贫困人口47.5万，贫困发生率下降14个百分点。完成79个片区连片扶贫开发，有效缓解了区域贫困、整体贫困;生产和就业扶持5.99万人、生态扶贫和移民搬迁安置5.94万人、低保政策兜底9.66万人、医疗救助5.71万人、灾后重建帮扶0.65万人、教育资助20.5万人次，首批215个贫困村顺利退出、9.1万人如期脱贫。土地政策扶贫、财政涉农资金整合、易地扶贫搬迁、教育、医疗、金融、产业等精准扶贫做法受到国家部委和省委省政府肯定并在全省推广，精准脱贫首战告捷。

　　社会建设全面加强。五年里，我们深度破解民生难题，全力抓好民生工程和民生实事，各项社会事业取得重要进展，城乡公共服务均等化水平显著提高。全市累计完成民生投入783亿元、占公共预算总支出72.8%;解决了城乡45万户居民住房困难、180万人安全饮水、50万人“行路难”问题，城镇新增就业15.4万人;第一所高校建成招生，建成2所全国中职改革示范校，新(改)建校舍220万平方米，学校D级危房全部消除，教育质量稳步提高;医疗卫生服务体系不断健全，服务水平不断提高，市中心医院成功创建三甲医院，二孩政策全面实施;米仓古道(蜀道)列入世界遗产预备名录，川陕苏区红军烈士陵园修缮开园，光雾山·诺水河国家地质公园揭牌开园，《挂印知县》《莲花开》等文艺精品获得国家、省级大奖;环中国自行车赛、四川国际自驾游交易博览会、巴人文化艺术节成功举办，全国双拥模范城、国家农业科技园区、“中国石刻艺术之乡”成功创建，国家卫生城市加快创建，获得全国文明城市提名和多个省级城市创建命名，增强了巴中的影响力。安全生产、食品药品监管持续加强，“平安巴中”建设扎实推进，一批老难信访问题有效化解，社会大局和谐稳定。国防动员、统计、保密、档案、地方志、民族、宗教、外事、台侨、妇女儿童、关心下一代、残疾人、老龄、气象和人防等工作都取得了可喜成绩。

　　生态优势持续巩固。五年里，我们始终牢记“绿水青山就是金山银山”，坚持绿色、低碳、高效发展，守住了四季绿水青山、常年蓝天白云、雨后云雾缭绕、终日鸟语花香的美好家园。全市森林覆盖率达到58%，城市环境空气质量优良天数达到90%，城市生活污水处理率、生活垃圾无害化处理率分别提高7个和8个百分点;国家环保模范城市创建加快推进，南江被列入国家首批主体功能试点示范县，3个县通过省级生态县验收或技术核查，7个乡镇获得国家级生态乡镇命名，生活垃圾焚烧发电厂建成投运;耕地红线得到有效保护，地质灾害防治、中小河流域治理、退耕还林工作成效明显。

　　改革开放成果丰硕。五年里，我们扎实推进重点领域和关键环节改革，打开山门对外大开放，敞开心门思想大解放，发展动力显著增强，市场活力不断迸发。39项全国全省改革试点完成阶段性目标，56个专项改革方案落地见效;区划调整加快推进，35个乡撤乡设镇，新增设7个街道办事处;市县两级智慧政务平台全面建成，基本实现“行权事项进平台、平台之外无权力”;先照后证、“五证合一”、“三证整合”等改革措施深入实施，催生市场主体8.16万户;新引进金融机构36家，设立“十大基金”，促进了财政金融支持发展方式转变;农村产权制度改革、城乡建设用地增减挂钩、公共资源交易、居民养老和医疗保险等改革走在全省前列，集团化办学取得重大进展，公立医院综合改革和分级诊疗深入推进。开放合作富有成效，新增友好城市6个，引进500强企业、央企和行业知名企业20家，累计到位招商资金1734.9亿元，是上个五年的13.8倍。

　　五年来，我们高度重视政府自身建设。努力建设法治政府，严格执行重大行政决策法定程序，依法制定规范性文件，坚持会前学法、政府法律顾问制度，落实行政执法责任制，主动接受人大、政协和司法监督。努力建设诚信政府，出台政府投资项目、公共资源交易、购买公共服务等制度规范，扎紧扎实制度笼子，严格依法履行协议、兑现承诺，以实际行动取信于民、取信于企。努力建设开明政府，网上公开征集民生难事、急事、要事，开设“政务访谈·市长在线访谈”，开通政府系统领导干部个人微信公众号，敞开胸怀吸纳民意，尽心竭力解决群众困难。努力建设高效政府，深入推进行政权力依法规范公开运行，优化政务服务流程，加强政务督查、效能监察和审计监督，深入治理行政乱作为和慢作为，促进政府廉洁高效运转。

　　各位代表，刚刚过去的2016年是本届政府任期届满之年。这一年，我们精准发力稳增长，深度研判经济形势，及时出台“1+5”政策组合拳，确保经济持续向好，多项经济指标增速进入全省前三，地区生产总值预计增长7.6%，全社会固定资产投资增长16.5%，工业投资增长19.2%，金融机构存款余额突破千亿元大关。我们着眼长远强基础，开工了巴万高速公路、黄石盘水库等一批重大项目，汉(中)巴(中)南(充)铁路进入国家中长期路网规划，完成省市重点项目投资800亿元。我们立足资源育产业，完成食品饮料、生物医药、石墨新材料、机械制造四大产业发展规划，引进好彩头、三强中药配方颗粒、10万吨石墨采选等一批重点成长型产业项目，成功创建全国新农村综合标准化示范市、全省农产品质量安全监管示范市和7个AAAA级旅游景区。我们聚焦难点惠民生，圆满完成省十项民生工程、20件民生大事和自选10件民生实事，一般公共预算民生投入194.5亿元。特别是去年5月，我们向全市人民承诺抓好公开征集的“7件民生难事、急事、实事”，目前已启动建设城区公办幼儿园8所，整治城区背街小巷98条，新增城区公共停车泊位1万个，新建乡镇污水处理站40个、乡镇加油站20座，改造农村危房2.38万户，对40个乡镇、320个村庄的垃圾进行了治理，承诺事项全部兑现。我们创新突破抓落实，川陕革命老区振兴发展规划获得国务院批复，25个重大项目正在做实前期、加快争取;相对集中行政许可权改革进入全国试点市行列;连片扶贫开发模式被国务院扶贫办确定为典型案例，土地增减挂钩政策支持扶贫攻坚的做法得到国务院肯定，叠加三大国土项目的经验在全国推广。党中央国务院、省委省政府和市委的一系列决策部署都不折不扣地落地生根、开花结果。

　　五年成绩的取得，标志着本届政府履职圆满收官，全市人民在全面小康的征途上迈出了新的一步。在推进全市经济社会又好又快发展的实践中，我们深刻体会到：必须坚持党的领导，旗帜鲜明地贯彻落实中央和省委的决策部署，在市委的坚强领导下，心无旁骛抓发展，凝心聚力搞建设。必须保持加快发展的定力，始终扭住目标、直面困难，与时俱进推动后发赶超，巩固扩大发展速度快于全国全省的态势。必须坚守民生至上的取向，始终不忘初心，不断增进民生福祉，让人民群众更多更好地共享发展成果。必须遵循改革开放的路径，以改革破解发展难题，以开放集聚发展要素，全面增强发展的动力活力。

　　各位代表，上述成绩的取得，是全市广大干部群众凝心聚力、奋勇拼搏的结果。在此，我代表市人民政府，向全市人民，向人大代表、政协委员、各人民团体、各民主党派、工商联、无党派人士，向所有关心支持巴中改革发展的同志们、朋友们表示衷心的感谢!向驻巴部队、武警官兵和民兵预备役人员，致以崇高的敬意!

　　成绩有目共睹，但困难和问题也不容忽视：经济总量小，人均水平低，发展层次不高;产业发展滞后，工业短板明显，支撑能力不足;互联互通的交通网络尚未形成，城镇化水平偏低，综合承载能力不强;脱贫攻坚任务重，公共服务欠账多，民生改善需要加力;少数干部政策敏感度不高，适应新常态、创新创造的能力有待加强，不想为、不敢为、不会为、不作为现象客观存在，政府行政效能有待提升。这些必须引起我们高度重视，在今后工作中切实加以解决。

　　今后五年主要目标任务

　　今后五年是我市后发赶超、脱贫攻坚、振兴发展的重要机遇期。面对更加复杂的经济形势、竞相发展的区域态势、异常繁重的脱贫任务、日益增长的群众需求，我们必须准确把握新形势、明确新任务、落实新举措，走出一条转型发展、后发赶超的新路子。

　　今后五年政府工作总的要求是：高举中国特色社会主义伟大旗帜，认真贯彻党的十八大和习近平总书记系列重要讲话精神，牢固树立“五大发展理念”，深入实施省委“三大发展战略”，全面落实市第四次党代会各项决策部署，坚持以立体交通为先导、以绿色农业为基础、以新型工业为支撑、以生态旅游为拓展、以全面小康为目标，统筹推进经济建设、政治建设、文化建设、社会建设和生态文明建设，决胜脱贫攻坚，建设“五彩巴中”，同步全面建成小康社会。

　　今后五年主要奋斗目标是：建设“五彩巴中”，推动区域交通节点向区域综合交通枢纽跨越、传统农业向现代农业跨越、工业弱市向工业强市跨越、文旅资源向文旅经济跨越、区域整体贫困向同步全面小康跨越，初步形成以绿色生态为核心竞争力的新兴增长极，全面完成市第四次党代会确定的各项目标任务。为此，我们将着力抓好事关全局的关键性工作。

　　着力做实精准脱贫。脱贫奔康是“五彩巴中”建设的底部基础。我们将进一步强化“唯此为大”“念兹在兹”的思想认识，围绕市委“四年集中攻坚、一年巩固提升”总体部署，聚焦“一本账、两不愁、三保障、四个好”，做细做实“六个精准”，抓好“五个一批”，实施精准脱贫与区域开发双轮驱动，到2019年底5县(区)全部摘帽、484个贫困村全部销号、22.86万贫困人口越过扶贫标准线，到2020年同步全面建成小康社会。巩固提升已有成果，创新工作推进、资源整合、群众参与、社会扶持等机制，形成较完备、可复制、能推广的脱贫攻坚制度体系。鲜明突出“以人为中心”的脱贫导向，把各项扶贫政策落实到基层、惠及到个人，使贫困群众有更多获得感和幸福感，建成“全国精准脱贫示范区”。

　　着力做优基础设施。基础设施是“五彩巴中”建设的先决条件。我们将以立体交通为先导，着眼互联互通、互利共赢，加快建设适度超前、覆盖城乡、功能配套、安全高效的现代基础设施体系。构建以“148”为骨架的“空铁公”立体交通体系，巴中恩阳机场通航，开通巴中至达州动车，建成巴(中)陕(西)、巴(中)万(源)高速公路和诺水河至光雾山一级公路，开工通江和南江通用机场、巴(中)南(充)快速铁路、苍(溪)巴(中)高速公路、镇(巴)广(安)高速公路巴中段，改造普通国省干线550公里，争取广(元)巴(中)铁路技改扩能、巴中至汉中快速铁路立项，推动巴城轨道交通建设，加快营(山)平(昌)万(源)高速公路前期工作。构建覆盖全域的综合水务体系，建成红鱼洞、黄石盘大型水库，开工建设8座大中型水库，解决农村70万人饮水安全问题。构建稳定共享的能源信息网络，建成一批110千伏及以上输变电站，抓好农网升级改造和智能电网建设，城镇燃气覆盖率100%，4G移动网覆盖城乡，村村通宽带，户户通广播电视。

　　着力做强产业支撑。产业是“五彩巴中”建设的核心支撑。我们将全力推进绿色价值再造，坚持全域全景全产业链、生产生活生态相融，加快构建三产联动的绿色产业体系。大力发展绿色农业，持续推进“三百工程”，新建现代农业标准化基地50万亩、生态养殖场(园)300个，建成农业主题公园15个、休闲农庄130个，创建省级现代农业示范园区10个，打造一批全国知名绿色有机农产品品牌。积极发展新型工业，重点打造500亿元食品饮料产业、100亿元生物医药产业、100亿元新能源新材料产业，因地制宜发展机械制造、建材家居、纺织服装产业，2020年工业增加值占GDP比重35%以上。突破发展现代服务业，成功申报米仓古道(蜀道)世界自然文化遗产，创建光雾山·诺水河世界地质公园，建成3个AAAAA级旅游景区、5个国家水利风景区、1个国家野生动物园、1个国家彩色植物园、5个省级以上森林康养基地，打造国际生态旅游和森林康养目的地，文旅产业对GDP贡献率20%以上;大力发展金融服务，建成秦巴金融街，强化保险保障，做优增值服务，用好证券助推脱贫攻坚政策，加快上市企业培育，支持2-3家企业成功上市，大力培育营销企业，让巴中产品在市场上有占有率、有附加值;建设10个省级现代服务业集聚区，打造年交易额超50亿元商贸物流园1个、10亿元物流园8个，建成川东北重要商贸物流中心。

　　着力做靓城市环境。城市是“五彩巴中”建设的直接载体。围绕建设现代森林公园城市，我们将持续以人的城镇化为核心，通过以绿兴城、以文兴城、以游兴城、以产兴城、以彩靓城，加快构建现代生态新型城镇体系。坚持城市新区、产业园区、旅游景区三区同建，加快推动新城崛起，打造宜居宜业环境。加大老城疏解力度，推进棚户区改造，完善配套设施，提升城市品质。实施九大绿化行动和增绿添彩工程，按照步步见绿、百米见景、五百米见园、千米见水的标准，打造一批绿廊绿道、人文景观、山地公园、湿地公园、景观水系，2020年实现城市绿化率45%以上、城市建成区绿地率38%以上、中心城区彩化率30%以上。启动历史文化名城创建，加大名镇(街区)、名村和传统村落保护利用力度，打造一批特色集镇和中心村，促进农民就地就近城镇化，2020年实现全市城镇建成区面积160平方公里、人口160万、城镇化率46%。

　　着力做好民生改善。改善民生是“五彩巴中”建设的终极目的。我们将牢牢把人民政府为人民的责任扛在肩上，最大限度为人民办实事、解难题、谋利益。抓好抓实各项民生工程，集中办好一批人民群众反映强烈的难事、急事、要事，民生投入占公共财政支出不低于70%。完善城乡公共服务，积极发展学前教育，均衡发展义务教育，优质发展高中阶段教育，大力发展职业教育，新建成一批全省示范学校，力争新建1所本科院校;推进“健康巴中”建设，基层医疗卫生实现阵地标准化、人员专业化、管理一体化，卫生事业达到全省先进水平;打造一批文化精品，培育一批文化名片，推动文化事业繁荣发展。加大社会共治力度，营造公平、正义、文明、法治的社会环境，为街道、社区、村组加强社会建设提供条件;传承弘扬笃学、朴实、勤劳、尚美的巴中人文精神，构筑向上向善的精神家园。实施严格的生态环境保护和问责制度，划定并严守生态保护红线，成功创建全国文明城市、国家卫生城市、国家环保模范城市、国家森林城市、国家园林城市、国家级生态文明建设示范市、红色历史文化名城。推进军民深度融合发展，提升优抚对象幸福指数，提高社会保障和防灾减灾水平，做好群众信访、安全生产和食品药品监管工作，严厉打击各种违法犯罪活动，确保社会和谐稳定。

　　着力做深改革开放。改革开放是“五彩巴中”建设的不竭动力。我们将坚持改革与开放良性互促，巧干实干、借力发展，激发后发赶超、振兴发展的动力活力。统筹推进国家、省改革政策举措落地落实，加快建设川陕革命老区综合改革试验区，争取重大改革事项在巴中试点，形成老区振兴发展的示范效应。深化“放管服”，加快互联网+政务服务改革，切实转变政府职能，为企业松绑，让市场发力。深化财政金融和投融资改革，组建巴中城市商业银行，加快培育多层次资本市场，拓宽融资渠道，大力发展直接融资，提高基金使用效益。坚持创新驱动发展战略，推动大众创业万众创新，实施民营经济市场主体增长计划，建成国家级经开区、国家级科技企业孵化器、省级高新技术产业园区。深化友城协作和市厅(校企)合作，围绕资源优势和主导产业推进全产业链招商，力争五年到位招商资金2500亿元以上。

　　2017年工作安排

　　今年政府工作总体要求是：全面贯彻党的十八大和中央、省委经济工作会议及市第四次党代会精神，牢固树立和贯彻落实新发展理念，围绕“建设五彩巴中、推动五大跨越”奋斗目标，坚持稳中求进工作总基调，以提高经济增长质量和效益为中心，坚定不移稳增长、促改革、调结构、惠民生、防风险，促进经济平稳健康发展和社会和谐稳定。

　　综合宏观经济形势和巴中发展实际，今年主要预期目标是：地区生产总值增长7.5%以上，全社会固定资产投资增长14%，规模以上工业增加值增长10%，社会消费品零售总额增长13%，地方一般公共预算收入增长10%，城乡居民人均可支配收入分别增长9.5%、11%，城镇登记失业率控制在4.5%以内。

　　为实现上述目标，今年重点抓好以下工作：

　　一、千方百计稳定经济增长

　　努力扩大有效投资。实施省市重点项目290个，完成年度投资855亿元以上，新开工项目151个，竣工项目67个。交通建设上，恩阳机场、巴(中)陕(西)高速年底前建成投运，加快建设巴(中)万(源)高速、巴城南环线，开工北环线东段、诺水河至光雾山一级公路，大力推进通江、南江通用机场和镇(巴)广(安)高速公路前期工作。水利建设上，加快推进红鱼洞、黄石盘、湾潭河等7座水库和市第二水厂、3个县城水厂扩容工程建设，力争开工青龙嘴、官房沟水库，积极推进江家口、青峪口、干沟河等14座水库和市第三水厂扩容等前期工作。电力通信建设上，开工城西220千伏、周家坝110千伏等输变电工程，建成4G基站800个，实施宽带乡村项目290个。项目储备争取上，做深做细做实项目前期工作，编制一批支撑长远发展的重大基础设施和产业发展项目，争取中央和省预算内资金20亿元以上、国家专项建设基金20亿元以上。

　　积极发展新型工业。抓好工业项目推进，实施重点工业项目80个，完成工业投资248亿元以上，新开工石墨高纯深加工、尖山300万吨石墨采选、通江风力发电等项目30个，竣工投产三强中药配方颗粒、好彩头食品生产基地(一期)、高性能碳素石墨(二期)、芦笋冷链物流及深加工等项目20个。实施企业培育行动，加快培育企业家队伍，旗帜鲜明地构建“亲”“清”政商关系，积极主动为企业排忧解难，为企业家合法经营撑腰壮胆。新培育规上工业企业30户、小微企业80户、上市后备企业3家。做强做大工业园区，推动巴中经开区争创国家级经开区和省级新型工业化产业示范基地、平昌星光工业园争创省级开发区、通江工业园创建省级绿色食品工业园，加快秦巴现代高科中医药、柳林食品、经开区石墨、平昌白酒、南江新材料等专业园区建设，力争工业集中度达到68%、同比提高2个百分点。

　　持续培育绿色农业。推进农业规模化发展，划定并严格保护永久基本农田，新建优质粮油基地6万亩、现代农业产业基地18万亩、畜禽标准化养殖场(协会、基地)50个、生态水产养殖园区10个、种养循环示范园区5个、巴城“菜篮子”基地2万亩，创建省级现代农业园区2个，建设农业主题公园3个、休闲农庄20个。增强新型经营主体带动能力，加大招引、回引力度，落实支农惠农政策，鼓励支持工商资本下乡，大力扶持涉农企业，全面提升新型业主的政治、经济、社会影响力，新培育市级以上农业产业化龙头企业5家、农民专业合作社100个、家庭农场50个、专业大户1000户，培训新型职业农民5000人;引导农户自愿以土地经营权、农具设施等入股龙头企业和农民专业合作社，采取“保底收益+按股分红”“互联网+农业”直供直销等方式，让农户分享加工销售环节的收益，农业产业化龙头企业及农民专业合作社带动农户面达到60%。加强特色农产品品牌培育，抓好通江银耳、巴山土猪、南江黄羊、大罗黄花、平昌青花椒、恩阳芦笋等特色农产品品质认证，建立生产、加工、营销标准体系，完善全程可追溯、互联共享信息平台，提升巴食巴适等品牌效益和市场美誉度。

　　加快提升现代服务业。统筹推进旅游业发展，编制《巴中市旅游业发展总体规划》，改革旅游管理体制，组建市文化旅游投资集团;启动全域旅游示范区创建，加快创建光雾山AAAAA级旅游景区、光雾山·诺水河世界地质公园和国家旅游度假区;凸显“红绿古特”优势，强化特色景区、特种景区建设，增加体验性和感染力;优化旅游线路，强化旅游宣传营销和旅游商品、旅游产品融合开发，继续办好光雾山红叶节、通江银耳节和省市乡村文化旅游节等特色节会，力争接待游客2600万人次以上，实现旅游收入210亿元。推动森林康养产业高点起步，完成“1+6”森林康养规划体系，建设省级以上森林康养基地2个、市级森林康养基地5个、县(区)级森林康养精品点(村)20个。推进金融产业集群集聚，引进设立各类金融机构4家，筹建地方法人银行，启动秦巴金融街建设，推动1370亿元签约授信落地，力争贷款增长20%以上。大力发展商贸物流产业，开工建设秦巴区域农副产品交易中心、空港物流园，加快建设盘兴物流园区、回风北路等特色商业街区、柳津桥等专业市场，建成投运容邦国际等商业集聚区。大力开展市外促销活动，推销巴中产、巴中造产品。

　　二、全力以赴推进脱贫攻坚

　　扎实做好精准脱贫。深入落实“六个精准”，全面开展脱贫攻坚“回头看”，动态调整脱贫对象，落实后续扶持政策，确保贫困对象稳定脱贫。对照贫困县“一低三有”、贫困村“一低七有”、贫困户“一超七有”标准，逐项落实达标措施，确保巴州区和恩阳区摘帽、215个贫困村销号、9.53万人脱贫。扎实抓好“五个一批”，扶持生产和就业5.27万人，移民搬迁安置4.39万人，医疗救助贫困人口全覆盖，因灾致贫群众得到及时救助，教育扶助政策全面落实，低保兜底应兜尽兜。积极推动“四好村”创建，以建设新农村、培育新农民、形成新风尚为重点，深化拓展“四好”内涵，细化完善创建标准，建成省级“四好村”320个、市级“四好村”350个。

　　有序实施连片开发。推动精准扶贫与区域开发双轮驱动，巩固提升79个已建片区成果，启动实施覆盖42个乡镇、503个村、81万人口的13个片区连片开发，确保建成一片、巩固一片、连接一片、发展一片。抓好开发项目实施建设，新建聚居点223个、巴山新居8219户、联网道路1400公里，配套完善文化、医疗、教育等公共服务设施，整理土地9.6万亩，发展特色产业基地137个，完成实用技术培训5.9万人次。推进区域协调发展，统筹用好项目资金，精准对接财政涉农资金项目库，兼顾非贫困村发展建设，有计划分年度整村推进、连片实施，破除基础设施、公共服务等瓶颈制约，提升片区整体发展水平。

　　切实强化脱贫保障。落实17个扶贫专项方案，细化年度目标任务，推动资金、项目、措施、责任“四个落实”。加大资金投入力度，实施扶贫投入预算管理，分村分户建立脱贫攻坚项目库，整合财政涉农项目44个、资金38亿元，撬动金融扶贫信贷投入150亿元，吸引社会投资20亿元以上。完善精准扶贫信息管理平台，充分发挥脱贫攻坚指挥中心功能，实现立体指挥、精准研判、节点推进、动态监管。创新脱贫攻坚机制，记好“一本账”，推进“六个盘活”，增强新型经营主体带动能力，推广财政投资股权量化和收益扶贫模式，完善入户督导、项目资金监管、脱贫验收、责任倒查等推进办法，将创新成果固化于制、形成长效。

　　三、全面有序统筹城乡建设

　　综合提升城市功能。优化城市规划设计，有序推进市、县“多规合一”，完善“一控规两导则”空间规划管控体系，优化提升《巴中市绿地系统规划》，完成3县绿地系统专项规划、3区20个绿化彩化景观方案。实施增绿添彩工程，推进巴城4大入城节点、城市干道、中心广场、沿河两岸的绿化彩化景观打造，实施环线、快速通道高截坡生态修复，加快莲花山、九寨山、义阳山等5个山地公园和兴文河心、津桥湖等5个湿地公园建设，巴城建成区新增绿地面积2万平方米、彩化面积6万平方米。实施厚植文化工程，高品位打造或提升八角楼、川博红色景点，保护提升南龛石窟、魁星阁、回风亭等历史遗迹。新建一批文化影院、体育场馆、心理驿站。加快市政设施建设，完成麻柳湾至巴河大桥滨河路建设，形成环滨路，建成巴城南龛大道、兴合大道(二期)、天星大道、张家河滨河路、黄家沟至插旗山连接线等市政道路;启动20公里地下综合管廊建设，推进魁星楼、回风东路等片区危旧房棚户区改造;加快经开区龙兴大道、通州大道，恩阳区14条城市主次干道和“五大组团”建设，同步推进3个县城建设。深入实施“五创联动”，成功创建国家卫生城市、森林城市，加强城乡环境综合治理，开展背街小巷、农贸市场、城郊结合部等重点部位和关键节点治乱治污，依法拆违12万平方米。

　　努力推动镇村融合。统筹推进镇村规划建设，完成10个重点镇城镇设计、10个新增国家级传统村落保护规划编制，坚持整镇整村推进，加快24个重点镇建设，建成中心村21个、聚居点200个、幸福美丽新村325个。积极推动产镇(村)一体发展，加快培育乡村旅游、森林康养产业，壮大农产品基地，完善初加工，建设10个集生产生活生态于一体的精品村。搞好教育、医疗、文化和养老等城乡基本公共服务综合配套。

　　稳步发展房地产建筑业。加快房地产去库存，认真执行房九条，严格商业和住宅用地管理，落实税收优惠政策，兑现购房补贴，用好用活住房公积金;打通公租房、安置房、商品房转换渠道，推进棚改和拆迁还房货币化安置;加快户籍制度改革，保障进城农民工依法享有居住地义务教育、就业、医疗等基本公共服务，鼓励农民进城购房;积极培育房屋租赁市场，商品房库存面积不断下降。引导房地产业转型发展，鼓励改善型住房建设，优化提升城市小区;鼓励房地产企业投资康养、旅游、度假、文体等新兴产业。加大建筑业培育力度，鼓励有条件的本地建筑企业向交通、电力、水利等国家重点投向领域拓展，力争年内新增资质和升级增项建筑房地产企业20家，实现建筑业总产值470亿元。深化绿色建筑行动，发展生态绿色建筑72万平方米，推广装配式建筑。

　　四、用心用情抓好民生事业

　　认真办好民生实事。解决百姓安居问题，新建农村安全住房2.63万套，搬迁8.99万人;改造农村危旧土坯房2.4万户;启动城镇危旧房棚户区改造1.55万户;千方百计加快安置还房建设进度。抓好乡村道路建设，实施农村公路改善、村道加宽、延伸联网工程1000公里。推进安全饮水工程，新建改造农村集中供水工程644处，新解决16万农村居民饮水安全;新建污水干管30公里。加快农村电网改造，完成239个村农网改造升级，解决南江县小水电供区40个村用电难问题。提升居民健康养老水平，加快建设市精神病康复院，二级以上公立医院开设老年病科，市残疾人康复中心全面投运，建成市惠民帮扶中心、县(区)残疾人康复中心和托养中心、51个城乡社区日间照料中心，新增养老床位1800张。实施全民参保登记计划，提高社会保险参保率。持续抓好生态环境质量改善，打好污染防治“三大战役”，强化生态环境监管执法，落实“河长制”，启动国家级生态文明示范市创建，建成4个省级生态县(区)。

　　大力发展社会事业。突出教育优先发展地位，推进城区公办幼儿园和义务教育学校建设，有效化解城市超大班，改造义务教育薄弱学校校舍8万平方米，建设农村教师周转房1300套;新建成“优质资源班班通”教室800个;新设置一批地方经济社会发展急需的专业，增强职业教育培训针对性;实施特岗计划和免费师范生计划，完成教师培训1.5万人次，稳定农村教师队伍。提升卫生计生服务水平，推进现有医疗机构提档升级，建成市中心医院南坝院区(一期)、市第一人民医院、市儿童医院、成都中医大附属医院巴中分院等医卫项目，建好市、县(区)人口健康信息平台，落实免费婚检孕检、新生儿疾病筛查等出生缺陷干预政策，创建“全国中医药文化宣传教育基地”。

　　积极繁荣文化事业。实施文化建设“四大工程”，培养一批文化专业人才、推出一批文化精品、挖掘一批民俗文化、打造一批文化名片，为“诗意山水，五彩巴中”提供坚实的文化支撑。完善文化服务体系，推动省现代公共文化服务体系示范县创建，加快秦巴文化活动中心、市体育中心和恩阳图书馆等重点项目建设，打通望王山运动公园环线，建成南龛文化产业园核心区，新建幸福美丽新村文化院坝180个。举办各类文体活动，继续办好巴人文化艺术节、川陕甘渝晋“百馆联动”和国际山地马拉松、环中国自行车赛等活动，积极开展送文化下基层活动，丰富群众文化生活。抓好文化传承保护，完成蜀道申遗巴中片区保护管理及环境综合整治规划，启动历史博物馆建设，完成革命旧址现状调查。

　　切实加强社会治理。加快法治巴中建设，深入开展依法治市“五大工程”和“七五”普法，行使好地方立法权，制定与经济社会发展相适应的政府规章，健全社会治安防控体系，规范信访秩序，依法解决群众诉求、化解矛盾纠纷。推动基层多元共治，促进基层民主协商，支持群团及各类社会组织参与社会治理，加强对困境儿童和农村留守儿童、妇女、老人、残疾人的关爱服务。强化安全监督管理，树牢大安全观，健全监测预警应急机制，加强安全基础设施和防灾减灾能力建设;规范企业安全管理，加强设施设备、作业现场、操作过程等环节监管;强化道路交通、建筑施工综合整治和消防、煤矿、非煤矿山、食品药品、大型活动监督管理，提升地质灾害防治能力，坚决遏制重特大安全事故发生;开展放心舒心消费城市创建，提高消费环境安全度、经营者诚信度、消费者满意度。

　　五、坚定不移扩大改革开放

　　深入推进重点领域改革。深化农村综合改革，完成巴州区全国第二批农村综合改革试点，建立市、县(区)农村产权流转服务中心，完善农村产权评估交易收储体系，有效盘活农村资产资源;推进农村集体资产和财政扶持资金股权量化、土地增减挂钩、农村房地一体不动产登记改革，完成国有林场、供销合作社等综合改革。推进财税体制改革，刚性执行财政政策，公开财政收支，全面提高财政绩效，优化财政产业发展投资引导基金管理，转变财政支持发展方式，最大限度发挥杠杆撬动作用;健全政府预算体系，盘活财政存量资金，强化政府债务管理和风险防控，推进政府向社会力量购买公共服务。强化投融资体制改革，启动3家县级农村信用联社改制，筹建通江银耳、南江黄羊交易中心;推广公私合营模式，引导社会资本参与公益性基础设施和配套服务建设。加快国资国企改革，健全公司法人治理结构，探索建立专职外部董事，推进产权多元化、资本证券化;通过兼并重组等方式妥善处理长期亏损、资产负债率高、停产半停产企业。统筹抓好其他领域改革，推动教科文卫、生态文明、城管执法及社会治理等改革持续见效。

　　务实开展对外合作。深化与周边地区的区域合作，在共同创建川陕革命老区综合改革试验区、旅游文化融合发展创新示范区、秦岭-大巴山国家公园、“三带三走廊”等方面迈出实质性步伐。深化拓展与“一带一路”及成渝西区域的经济合作，增强主动融入和承接产业转移能力。加强与发达地区的产业合作，联手开发石墨、天然气、巴药、绿色产品等优势资源，进一步拓展市场空间。拓展与高校、科研院所、企业的产学研合作，推动在巴设立研发机构、分公司、工作站，建立产学研联盟。

　　精准实施招商引资。明确招商重点方向，抓住把准经济发展新方位，围绕食品饮料、生物医药、新能源新材料、文化旅游、森林康养、优质教育、有机特色农业等产业深入论证、精细包装，推出招商项目300个。精准对接重点企业，推动年产3.5万吨天然石墨负极材料生产、汽车轻量化石墨烯改性塑料、农产品电商物流园、光雾山旅游综合开发等项目签约落地，引进重大产业项目25个以上，各类招商项目到位资金530亿元以上。真情抓好投资服务，组建企业投资促进协调服务中心，推行项目专员代办制，开好企业协调服务座谈会，点对点解决企业困难和问题，坚决整治损害投资软环境行为。

　　六、集智聚力推进创新创业

　　积极搭建“双创”平台。建强园区孵化基地，编制巴中市“双创”中心发展规划，加快国家农业科技园区建设，创建经开区省级高新技术产业园区，建成巴中置信省级科技企业孵化器，培育普瑞制药等5家国家高新技术企业、秦巴电商产业园等2家省级大学生创新创业孵化园，新增孵化面积2万平方米以上。推进创新载体建设，培育七彩林业、巴山牧业、秦巴云顶等3个省级工程技术研究中心(产业技术研究院)，组建石墨烯产业技术研究中心，建立万事康等5个市级以上院士(专家)工作站。健全服务指导机构，市、县(区)设立创业指导中心和服务窗口，组建创业指导专家团队，强化创业孵化和跟踪扶持，提高创业成功率和稳定经营率。

　　加快培育“双创”主体。吸引高端和实用人才创新创业，围绕“高精尖缺”引进一批金融专家、职业经理人、高级工程师等高层次人才，大力发挥实用技术人才和乡土专家作用，做强创新创业核心支撑。支持涉农科技人才引领创业，完善促进科技成果转化转移政策体系，允许符合条件的事业单位科技人员带着成果或项目领办创办企业。回引在外人士返乡创业，深入实施返乡创业五年行动计划，大力回引巴中籍企业家、农民工返乡创业兴业，力争回引创业3700人以上。推进大学生自主创业，加强大学生创新创业俱乐部建设，推动组建大学生创新创业联盟，充分激发青年群体创新创业热情。支持民营企业创新创业，制定实施《加快民营经济转型升级三年行动计划(2017-2019)》，推动民营企业建立现代企业制度，建成市县民营企业服务中心和投诉举报中心，新增民营经济市场主体2万户、科技企业50家。

　　持续优化“双创”环境。改进政务服务方式，深入推进“放管服”改革，深化相对集中行政许可权改革，推动智慧政务平台优化升级，全面推行先照后证、一照多址、一址多照、企业“五证合一”和个体工商户“三证整合”。着力强化财税支持，全面落实创新创业财税减免优惠政策，减轻税费负担，降低要素价格，降低创新创业成本;加大财政支持力度，推动产业扶贫资金向创新创业园区、厂房租赁、水电气路建设等方面倾斜;用活科技创新引导、返乡创业分险、创业贷款担保等基金，引导社会资金和10亿元金融资本参与支持“双创”工作。

　　加大保护激励力度，推进知识产权入股、股权期权奖励;落实创新创业杰出人才评价支持办法，用好安家补助、岗位激励、项目和平台支持等政策，营造大众创业万众创新的浓厚氛围。

　　各位代表，蓝图已经绘就，上述目标任务是我们对全市人民的庄严承诺，是我们的职责使命。我们将在市委坚强领导和市人大、市政协监督支持下，进一步加强政府自身建设，从脱贫攻坚最具体的问题抓起，从加快发展最重大的问题抓起，从安全稳定最现实的问题抓起，知难而进，勇于担当，奋力作为。始终把人民作为第一牵挂。落实好以人民为中心的发展思想，坚持一切为了人民、紧紧依靠人民，视人民群众为衣食父母。解民难、纾民忧、顺民意，人民群众期盼的事我们都要一件一件地做好，即使有困难也要给群众讲清楚，对群众一时不理解的要做好耐心细致的思想工作，让人民群众不断增进幸福感和获得感。始终把法纪作为第一准绳。我们的各项工作都必须把政治纪律和政治规矩挺在前面，牢固树立“四个意识”，在纷繁复杂的政务事务面前，时刻牢记宪法法律赋予我们的职责，明晰纪律规矩划定的底线，自觉依法依规依政策办事，坚持按事权规范请示报告工作，在急难险重面前敢于亮明身份、挺身而出，让政府工作蹄疾而步稳地在阳光法治轨道上运行。始终把效率作为第一生命。行政效率就是服务群众、促进发展、解决实际问题能力水平的直接体现，政府机关必须把党的路线方针政策内化于心、外化于行，熟知程序、规范操作，能办的立即办，暂时不能办的讲清应该怎么办，绝对不行的直接说明道理，保证不误人误事，做到雷厉风行、件件抓落实、事事有回音。始终把公信力作为第一形象。公信力就是执政形象，诚信政府必然带出诚信社会。摒弃简单包揽一切，结合巴中实际把需要和可能深度公开，绝不讲大话吓唬群众，绝不开空头支票糊弄群众。始终把廉洁作为第一底线。为民是政之情怀，廉洁乃官之底线，肩负“两个责任”，带头行为示范，是每个政府工作人员必须守住的基本节操。不断提升人生境界，牢固树立廉洁从政意识，存正心走正道做正事，绝不让歪风邪气有空子可钻，旗帜鲜明地查处不廉洁的事、处理不廉洁的人。健全正向激励机制，真正做到按本色做人、按角色做事，锻造忠诚干净担当的政府工作队伍。

　　各位代表，上任伊始，我承诺每年亲自督办几件老百姓最期盼解决的民生实事。经过梳理，今年承诺督办的事项为：新(改)建5所城区公办幼儿园，新建10000个城区停车泊位、14座城市公厕和41个乡镇污水处理站。

　　各位代表，五年又是一个新的征程。时代赋予我们后发赶超、脱贫攻坚、振兴发展的神圣使命，唯有团结才能汇聚力量，唯有进取才能惠及人民，唯有奋斗才能赢得未来。让我们更加紧密地团结在以习近平同志为核心的党中央周围，在省委、省政府和市委的坚强领导下，团结带领全市人民，积极开拓，务实作为，奋力推动“五个跨越”，为决胜脱贫攻坚、建设“五彩巴中”、同步全面建成小康社会而努力奋斗!
